# Assessment of Genetic Polymorphisms in the Rewa Population of Central India Using Y-Chromosomal STR Markers

**DOI:** 10.17691/stm2021.13.6.05

**Published:** 2021-12-28

**Authors:** A. Mishra, U. Gondhali, S. Mishra, S.K. Choudhary

**Affiliations:** Research Scholar, School of Forensic Science and Risk Management; Rashtriya Raksha University, Raksha Shakti Rd., Lavad, Gandhinagar, Gujarat, 382305, India;; Lecturer, Jindal Institute of Behavioural Sciences; O.P. Jindal Global University, Sonipat Narela Road, Near Jagdishpur Village, Sonipat, Haryana, 131001, India;; Intern; Indira Gandhi Institute of Medical Sciences, Allahabad bank, Bailey Rd., Sheikhpura, Patna, Bihar, 800014, India; Senior Assistant Professor, School of Forensic Science and Risk Management; Rashtriya Raksha University, Raksha Shakti Rd., Lavad, Gandhinagar, Gujarat, 382305, India;

**Keywords:** Y-chromosomal STR, Rewa population in Central India, haplotype diversity, phylogenetic analysis

## Abstract

**The aim of the study** was to analyze unrelated samples from the Rewa male population of Central India by targeting Y-short tandem repeats (STR) and then compare the results to previously published Y-STR haplotype data.

**Materials and Methods:**

A total of 181 unrelated male subjects from the Rewa population were genotyped for seventeen Y-STRs (DYS19, DYS389I, DYS389II, DYS390, DYS391, DYS392, DYS393, DYS385a/b, DYS437, DYS438, DYS439, DYS448, DYS456, DYS458, DYS635, and Y-GATA-H4) by using an AmpFlSTR® Yfiler™ multiplex kit (Thermo Fisher Scientific, USA). The allele frequencies and forensic parameters were evaluated.

**Results:**

A total of 111 distinct Y-STR alleles with corresponding frequencies ranging from 0.006 to 0.829 were identified. The gene diversity values ranged from 0.3092 at DYS437 to 0.8188 at DYS385b. The studied population showed a high level of haplotype diversity (0.9985) and discrimination capacity (0.927). A haplotype analysis was also conducted. Among the 181 unrelated male samples, 165 haplotypes and 153 unique haplotypes were found. Additionally, Rst (genetic distance) values were calculated using the analysis of molecular variance (AMOVA) for the studied population and for other 18 populations described in the literature. The Rst provides a convenient parameter for estimating the level of genetic differentiation from the microsatellite data. Based on these Rst values and using the multidimensional scaling plot, a neighbor-joining tree was constructed.

**Conclusion:**

The high values of haplotype diversity and discrimination capacity indicate a great potential for distinguishing between male individuals in the studied population. The present population data are expected to find their use in forensic caseworks and population genetics.

## Introduction

Rewa or Rewah district is located in the Vindhyan Range Plateau, in the North-Eastern part of Central India. According to the 2011 India Census, the total population size of Rewa is about 2.5 million; among them, there are 1.3 million males and 1.2 million females. The population density is 377 people per square kilometre area. Rewa was known as a princely state during the time of British Empire; today, its population is composed of various groups like tribes, casts, and communities living under various environmental conditions like forests, waterfalls, rivers, lush greenery, monuments, and forts (https://rewa.nic.in/).

Forensic laboratories across the globe are conducting Y-short tandem repeat (STR) typing for their routine casework analysis as well as population genetics studies [1, 2]. Y-STRs are short repeats of 2–7 base pairs and are widely spread throughout the Y-chromosome. Due to their high polymorphism, they are used as genetic markers in forensic analyses for anthropological cases, missing individuals, and disaster victim identification where only distant male relatives are available, as well as paternal-kinship analysis, population genetics, genealogical DNA testing, and human migration analysis. The Y-STR markers are highly valuable in cases of male/female cell admixture, namely in sexual assault cases. There, Y-STR has a significant role in differentiating between male and female DNA samples so as to produce interpretable results [3, 4]. Due to a lack of recombination process, Y-chromosome is considered as a male-specific marker and represents the paternal line inheritance with a little mutation or gene conversion [5, 6]. The non-recombination part of Y-chromosome DNA is useful in those cases where autosomal DNA profiling would be non-informative. Additionally, Y-STR haplotyping has been useful for solving motherless paternity cases by determining the patrilineal lineage of male perpetrators. Over time, various Y-STR multiplexes commercial kits with an ultra-high discrimination capacity between genetic variations have been developed. These characteristics of Y-chromosome make Y-STR profiling an important tool in forensic investigations, tracking human evolution, tracing bio-geographic ancestry of individuals, and detecting genetic differences between populations by identifying the informative Y-STR haplotypes [7–10].

So far, only a limited number of genetic studies on Y-STRs among the Central Indian population have been reported. Hence, there is a need to conduct more studies on this population [11–15].

**The aim of the study** was to analyze unrelated samples from the Rewa male population of Central India by targeting Y-short tandem repeats and then compare the results to previously published Y-STR haplotype data.

## Materials and Methods

### Sample collection and ethical consideration

**Figure 1.**
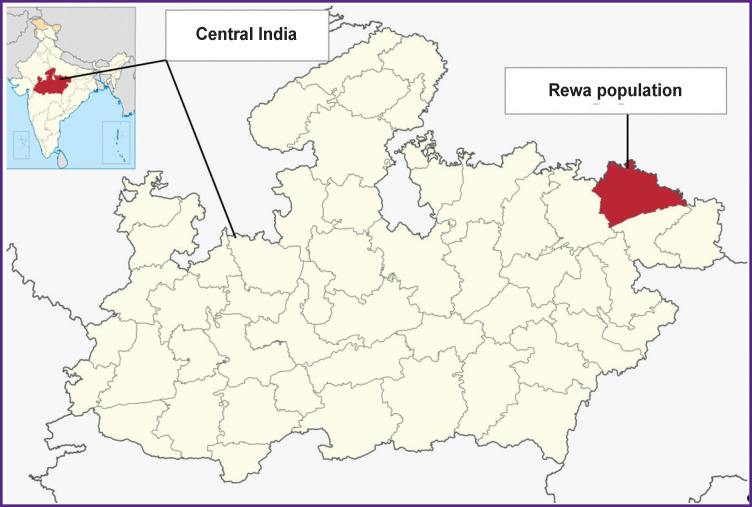
Map of India (top left corner) with Central India highlighted and the map of Central India (main picture) with the Rewa district highlighted

Prior to conducting this study, ethical clearance had been obtained from the Ethical Committee of the Raksha Shakti University (Gujarat, India). A total number of 181 unrelated male individuals’ blood samples were collected randomly for the purpose of this study. All the samples belong to the population of Rewa, a state in Central India (Figure 1). Peripheral blood from each individual was collected and stored in EDTA tubes. Individuals from these settlements were approached in person with the help of their relatives and friends. All the participants were briefed about the purpose of the study. An informed written consent was obtained from each participant, in accordance with the Declaration of Helsinki (2013). The study participants ranged from 20–50 years of age.

### DNA extraction and quantification

Phenolchloroform extraction was performed to extract genomic DNA from whole blood samples (n=181). The extracted DNA samples were quantified using a Quantifiler® Duo DNA Quantification Kit (Thermo Fisher Scientific, USA) in accordance with the manufacturer’s protocol. Quantified DNAs were normalized to the concentration of 1.0 ng/μl and then taken for PCR amplification.

### Amplification

DNA samples were subjected to the amplification by using seventeen Y-STR loci (DYS19, DYS389I, DYS389II, DYS390, DYS391, DYS392, DYS393, DYS385a/b, DYS437, DYS438, DYS439, DYS448, DYS456, DYS458, DYS635, and Y-GATA-H4). PCR conditions were set as per the manufacturer’s instructions. The total volume of the PCR reaction mixture comprised 25 μl of reagents (5.0 μl of primers mix, 10.0 μl of master mix, and 10.0 μl of nuclease-free water). The amplification was run on a GeneAmp PCR System 9700 Thermal Cycler (Applied Biosystems, USA) using an AmpFLSTR Yfiler™ PCR kit (Thermo Fisher Scientific). Positive and negative controls were also run throughout the system.

### DNA electrophoresis and analysis

The PCR products were size separated with the help of capillary electrophoresis using an ABI 3100 Genetic Analyzer (Life Technologies Corporation, USA) and size-characterized with the GeneScan 500 LIZ internal lane size standard (Thermo Fisher Scientific) as per the manufacturer’s recommended protocol. The GeneMapper ID-X Software Version 1.4 (Applied Biosystems) was used to determine the amplified fragments’ sizes. All alleles’ designations were based on a comparison with the allelic ladders provided by the AmpFLSTR Yfiler™ system (Thermo Fisher Scientific). The present study was carried out in accordance with the quality assurance standards recommended by the Scientific Working Group on DNA Analysis Methods (SWGDAM) [16].

### Statistical analysis

Forensic and population genetic parameters were evaluated for seventeen Y-STRs. The gene diversities (GD) of the analysed 17 Y-STRs were calculated according to Nei and Tajima [17]. The allele and haplotype frequencies were determined using the counting method. Haplotype diversity (HD) was calculated using the formula:


$$\text{HD=(N/N–1)(1–}\sum x^{2}),$$

where *x* is the (relative) haplotype frequency of each haplotype in the sample and *N* is the sample size. The polymorphism information content (PIC) was calculated via the marker allelic frequencies using the following equation:


$$\text{PIC=1 - }\sum_{i=1}^{n}p_{i}^{2}-\sum_{i=1}^{n-1}\sum_{j=i+1}^{n}2p_{i}^{2}p_{j}^{2},$$

where *n* is the number of alleles and *p_i_* is the allele probability of the *i^th^* allele. The power of discrimination (PD) was also calculated as 1–PM, where PM is the probability matching. All the above parameters were calculated using the online software STR Analysis for Forensics (STRAF) [18]. The discrimination capacity (DC) was calculated by using the formula: DC=*N*_d_/*N*_t_, where *N*_d_ denotes the number of unique haplotypes and *N*_t_ denotes the total number of haplotypes. We used the Haplotype Analysis v1.04 software [19] to characterize the haplotype. Using the analysis of molecular variance (AMOVA) [20], population pairwise genetic distances (Rst) and their corresponding p-values were calculated to reveal the differences between the studied population and other eighteen populations (described in the literature) and then estimate their genetic relationships, using the YHRD website (https://www.yhrd.org). The Rst is equivalent to the fraction of the total variance in allele size (in terms of number of repeat units) between populations and is given by: Rst=(*S–S*_w_)/*S*, where *S* is twice the variance in allele size over the populations and *S*_w_ is twice the average within-population variance in allele size. Reduced dimensionality spatial representation of the populations based on Rst values was performed using multidimensional scaling (MDS). The MEGA 6.0 software was used to construct the neighbor-joining tree with 1000 bootstrap replicates [21]. Neighbor-joining is another distance-based method for phylogenetic tree construction. It uses the clustering approach to constructing the tree.

## Results

### Y-chromosome diversity

Distribution of allele frequencies and gene diversity for the 17 Y-STR loci are presented in Supplement 1. A total of 111 distinct alleles with corresponding frequencies ranging from 0.006 to 0.829 were observed in the studied population. The mean value was found to be 6.529 alleles per locus. Loci DYS385b (n=10), DYS635 (n=9), and DYS389II, DYS390, DYS392, DYS385a, DYS458, DYS456 (n=8) have the highest number of alleles. Hence, the highest degree of polymorphism was observed with the maximum number of alleles. Among others, the DYS391, DYS437, and Y-GATA-H4 loci have the least number of alleles (n=4), indicating the lowest degree of polymorphism.

### Haplotypes analysis

A total number of 165 Y-haplotypes were obtained from the 181 individuals using the 17 Y-STR Yfiler™ kit. Among 165 haplotypes, the number of unique haplotypes was 153 (92.7%). The remaining 12 haplotypes (7.27%) were replicated between the subjects under study; 10 haplotypes were replicated twice; one haplotype was observed three times and another one was observed five times. The discriminating capacity of the 17 Y-STR Yfiler™ was found to be 0.9985. The overall haplotype diversity for the studied male population was found to be 0.9985. Both the discriminating power and haplotype diversity were remarkably high in the studied population (Supplement 2).

### Gene diversity and other forensic parameters

**Figure 2.**
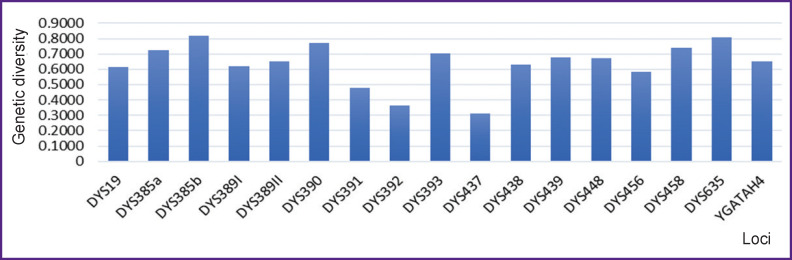
Genetic diversity for 17 single-locus Y-STRs in the Rewa population of Central India (n=181) Locus-wise genetic diversity values are presented with the maximum at DYS385b (0.8188) and the minimum at DYS437 (0.3092)

The values of GD for the seventeen Y-STR loci are shown in Figure 2. According to the estimated GD for each locus, the highest gene diversity was observed at loci DYS385b and DYS635 (GD was 0.8188 and 0.8055, respectively), whereas DYS437 and DYS392 loci demonstrated the lowest gene diversity values (GD was 0.3092 and 0.3664, respectively). Hence, the results showed a greater level of genetic polymorphism in fourteen Y-STR loci with the GD values exceeding 0.5, whereas three locus-possessed GD values were less than 0.5. The same trend was observed for the PIC values; the highest ones were observed at locus DYS385b and locus DYS635 (PIC was 0.7901 and 0.7729, respectively), whereas the DYS437 and DYS392 loci showed the lowest values (PIC was 0.2873 and 0.3395, respectively). In the probability matching test, DYS385b showed a lower value (0.1857), while DYS437 showed the highest one (0.6925) in this set of studied STRs. For the PD, DYS437 showed the lowest value (0.3075) while DYS385b demonstrated the highest one (0.8143). Gene diversity and other parameters which are of forensic interest are summarised in Table 1.

**Table 1:** Forensics-relevant parameters observed in the population of Rewa, India (n=181)

Locus	GD	PIC	PM	PD
DYS19	0.6127	0.5492	0.3907	0.6093
DYS385a	0.7225	0.6910	0.2815	0.7185
DYS385b	0.8188	0.7901	0.1857	0.8143
DYS389I	0.6184	0.5389	0.3851	0.6149
DYS389II	0.6502	0.5989	0.3534	0.6466
DYS390	0.7727	0.7362	0.2316	0.7684
DYS391	0.4779	0.3904	0.5247	0.4753
DYS392	0.3664	0.3395	0.6356	0.3644
DYS393	0.7050	0.6426	0.2989	0.7011
DYS437	0.3092	0.2873	0.6925	0.3075
DYS438	0.6297	0.5582	0.3738	0.6262
DYS439	0.6779	0.6143	0.3258	0.6742
DYS448	0.6708	0.6076	0.3329	0.6671
DYS456	0.5845	0.5398	0.4188	0.5812
DYS458	0.7389	0.6918	0.2652	0.7348
DYS635	0.8055	0.7729	0.1990	0.8010
Y-GATA-H4	0.6498	0.5775	0.3537	0.6463

Here: GD — gene diversity; PIC — polymorphism information content; PM — probability matching; PD — power of discrimination.

### Population statistics

**Figure 3.**
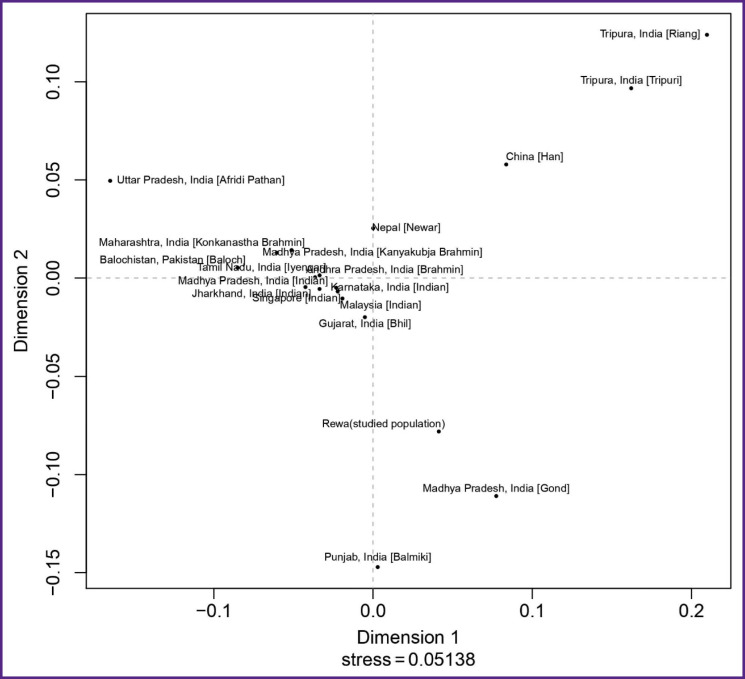
Multidimensional scaling (MDS) plot based on pairwise Rst values between the studied population and reference populations from the YHRD

**Figure 4.**
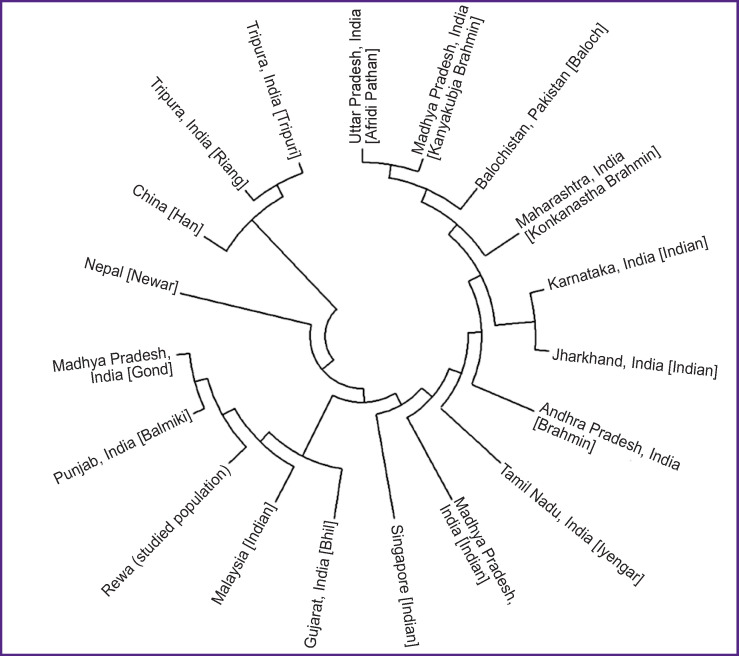
Neighbor-joining dendrogram showing the relationship of the Rewa population with the previously reported population data The tree is based on the pairwise Rst values between the studied population and other populations reported in the literature. The dendrogram was generated using the YHRD tool for seventeen Y-STRs loci common to all these populations (DYS19, DYS389I, DYS389II, DYS390, DYS391, DYS392, DYS393, DYS385a/b, DYS437, DYS438, DYS439, DYS448, DYS456, DYS458, DYS635, and Y-GATA-H4)

Rst is one of the most widely used measures for genetic differentiation; it plays a central role in ecological and evolutionary genetic studies. Using AMOVA, population Rst and their respective p-values (p<0.05) were calculated in order to understand the genetic relationship between the studied population and other eighteen populations characterized in the literature: China [Han]; Uttar Pradesh, India [Afridi Pathan]; Punjab, India [Balmiki]; Gujarat, India [Bhil]; Andhra Pradesh, India [Brahmin]; Karnataka, India [Indian]; Madhya Pradesh, India [Gond]; Jharkhand, India [Indian]; Madhya Pradesh, India [Indian]; Tamil Nadu, India [Iyengar]; Madhya Pradesh, India [Kanyakubja Brahmin]; Maharashtra, India [Konkanastha Brahmin]; Tripura, India [Riang]; Tripura, India [Tripuri]; Malaysia [Indian]; Nepal [Newar]; Balochistan, Pakistan [Baloch]; and Singapore [Indian] [2, 12, 22–28]. For the comparison, the same 17 Y-STR loci were used (Table 2) with the statistical significance determined by a permutation test with 10,000 replicates. The genetic distances (Rst) and their corresponding p-values for the eighteen populations are illustrated in Table 2. According to the Rst values, the Afridi Pathan population (0.3595) from Uttar Pradesh and the Riang tribe (0.2802) from Tripura showed the longest genetic distances, while the Gond population (0.0465) from Madhya Pradesh and the Bhil population (0.0609) from Gujarat demonstrated the shortest genetic distances. The comparisons between the studied population and those previously reported showed significant variations in the Rst value (p<0.05). The clustering pattern of studied population compared with other Indian populations is shown in the MDS plot generated from the population data (Figure 3). This finding is also illustrated by the clustering pattern observed in the neighbor-joining tree (Figure 4).

**Table 2:** Molecular variance of pairwise distances between the studied population and reference populations from the YHRD (based on Rst values)

Population	Rewa (studied population)	China [Han]	Uttar Pradesh, India [Afridi Pathan]	Punjab, India [Balmiki]	Gujarat, India [Bhil]	Andhra Pradesh, India [Brahmin]	Karnataka, India [Indian]	Madhya Pradesh, India [Gond]	Jharkhand, India [Indian]	Madhya Pradesh, India [Indian]	Tamil Nadu, India [Iyengar]	Madhya Pradesh, India [Kanyakubja Brahmin]	Maharashtra, India [Konkanastha Brahmin]	Tripura, India [Riang]	Tripura, India [Tripuri]	Malaysia [Indian]	Nepal [Newar]	Balochistan, Pakistan [Baloch]	Singapore [Indian]
Rewa (studied population)	*	0	0	0	0	0	0	0	0	0	0	0	0	0	0	0	0	0	0
China [Han]	0.1663	*	0	0	0	0	0	0	0	0	0	0	0	0	0	0	0	0	0
Uttar Pradesh, India [Afridi Pathan]	0.3595	0.2856	*	0	0	0.0012	0.0082	0	0.0032	0.0002	0.0115	0.0418	0.0093	0	0	0	0.0001	0.0007	0
Punjab, India [Balmiki]	0.077	0.2511	0.4424	*	0	0	0	0	0	0	0	0	0	0	0	0	0	0	0
Gujarat, India [Bhil]	0.0609	0.0995	0.2223	0.1299	*	0	0.0001	0	0.0001	0	0	0	0	0	0	0	0	0	0
Andhra Pradesh, India [Brahmin]	0.1222	0.1368	0.1244	0.148	0.0408	*	0.2226	0	0.0317	0.0918	0.2903	0.026	0.0509	0	0	0.0026	0.001	0	0.0151
Karnataka, India [Indian]	0.1272	0.1363	0.091	0.1692	0.0378	0.0032	*	0	0.2345	0.1014	0.0883	0.2651	0.0838	0	0	0.0001	0.001	0	0.0004
Madhya Pradesh, India [Gond]	0.0465	0.1625	0.4585	0.0863	0.1132	0.1597	0.1724	*	0	0	0	0	0	0	0	0	0	0	0
Jharkhand, India [Indian]	0.1215	0.1279	0.1114	0.1703	0.033	0.0125	0.0022	0.173	*	0.0731	0.04	0.0402	0.0111	0	0	0	0.0002	0	0
Madhya Pradesh, India [Indian]	0.0875	0.1249	0.1253	0.1178	0.0178	0.0041	0.004	0.126	0.0037	*	0.054	0.0007	0.0016	0	0	0	0	0	0
Tamil Nadu, India [Iyengar]	0.1325	0.1398	0.0921	0.1576	0.0445	0.0021	0.0106	0.169	0.0155	0.009	*	0.0478	0.0541	0	0	0.0111	0.011	0	0.0617
Madhya Pradesh, India [Kanyakubja Brahmin]	0.1843	0.1724	0.0511	0.2334	0.0797	0.0171	0.0025	0.249	0.0146	0.0235	0.0189	*	0.1552	0	0	0	0.0005	0	0
Maharashtra, India [Konkanastha Brahmin]	0.1618	0.1609	0.0972	0.1833	0.0699	0.0143	0.0117	0.217	0.0253	0.0233	0.0185	0.0076	*	0	0	0.0001	0.003	0	0.0009
Tripura, India [Riang]	0.2802	0.1667	0.5846	0.4268	0.2793	0.3314	0.3342	0.324	0.3219	0.2772	0.3061	0.3741	0.3308	*	0.0236	0	0	0	0
Tripura, India [Tripuri]	0.2054	0.0906	0.4449	0.3339	0.1784	0.2248	0.2282	0.226	0.2176	0.1935	0.1983	0.2661	0.2309	0.022	*	0	0	0	0
Malaysia [Indian]	0.0645	0.1208	0.1905	0.0766	0.0206	0.0167	0.0346	0.093	0.0309	0.0135	0.0189	0.0633	0.0396	0.2499	0.1682	*	0	0	0.0594
Nepal [Newar]	0.1308	0.1048	0.1959	0.2149	0.0501	0.0487	0.0477	0.181	0.0551	0.0456	0.0343	0.0632	0.0474	0.1978	0.1073	0.0477	*	0	0.0001
Balochista, Pakistan [Baloch]	0.2023	0.1597	0.0902	0.2241	0.0802	0.032	0.0422	0.24	0.049	0.0438	0.0369	0.0421	0.054	0.3717	0.2728	0.0664	0.096	*	0
Singapore [Indian]	0.0795	0.1061	0.1583	0.1062	0.017	0.0089	0.0239	0.111	0.023	0.0089	0.0081	0.046	0.03	0.2358	0.1555	0.0023	0.031	0.0463	*

* p-values are shown above the diagonal and Rst values below it.

## Discussion

Due to the absence of recombination process in the Y-STR chromosome markers, they are considered highly effective in studying the genetic diversity of male individuals in a population. In order to understand the origin of modern humans, it is important to conduct genetic studies of various populations. Although such studies have been reported from different regions of Central India, no population studies have been conducted and reported from the Rewa region.

The present study was conducted with 181 unrelated male individuals of the Rewa population whose Y-STR markers were determined, processed, and then compared with published data on other populations for the genetic distance or genetic relationships. According to the results, the DC value (0.9985) was higher and these seventeen Y-STRs had more polymorphism and higher systematic efficiency in the Rewa populations; these parameters can be used as a useful forensic marker set. The GD value exceeded 0.5 in fourteen Y-STR loci, which showed a higher level of polymorphism. Along with that, three loci had GD values of less than 0.5; that indicated a lower level of polymorphism, which is less suitable for forensic purposes in this population.

The pairwise comparison between the studied population and those reported by others demonstrate that the Rewa people have a close affinity to Madhya Pradesh, India [Gond], followed by Gujarat, India [Bhil] Malaysia [Indian], Punjab, India [Balmiki], Singapore [Indian], and Madhya Pradesh, India [Indian]. The studied population showed a greater genetic affinity with the population of Uttar Pradesh, India [Afridi Pathan]. As observed in the neighbor-joining tree, the Madhya Pradesh, India [Gond], Gujarat, India [Bhil] Malaysia [Indian], Punjab, India [Balmiki], and Madhya Pradesh, India [Indian] cluster belong to the same tree branch. The tree also revealed that in Central India, the populations of the same ethnic origin (Madhya Pradesh, India [Indian] and Madhya Pradesh, India [Kanyakubja Brahmin]) belong to different clusters. The authors encourage other researchers to explore more genetic polymorphism in the Rewa population.

## Conclusion

An attempt has been made to better understand the genetic relationship between Rewa (Central India) and other neighbouring populations. To this end, 181 Rewa male individuals were genotyped using an AmpFlSTR® Yfiler™ multiplex kit. This is the first report that provides population-based data on genetic variations and Y-STR polymorphism in the Rewa population using seventeen Y-STR markers. Here, we also report on the 17 Y-STR loci allelic frequencies and haplotype distribution in the Rewa population, which exhibit a significant discrimination capability, which can be applied to forensic casework and population studies.

## Figures and Tables

**Table:** Allele frequencies for 17 single-locus Y-STRs in the Rewa population (n=181)

Allele	DYS19	DYS389I	DYS389II	DYS390	DYS391	DYS392	DYS393	DYS385a	DYS385b	DYS438	DYS439	DYS437	DYS448	DYS458	DYS456	DYS635	Y-GATA-H4
8										0.006							
9					0.028					0.337							
10					0.652	0.044		0.006		0.492	0.116						0.039
11		0.022			0.315	0.785	0.006	0.088		0.133	0.392						0.442
12		0.116				0.022	0.354	0.072		0.028	0.387	0.006					0.365
13	0.006	0.475				0.133	0.320	0.155	0.006		0.094				0.028		0.155
14	0.127	0.381				0.006	0.260	0.099	0.077	0.006	0.011	0.823		0.028	0.050		
15	0.541	0.006			0.006	0.006	0.061	0.475	0.072			0.111		0.033	0.602		
16	0.282					0.006		0.094	0.282			0.050		0.188	0.221		
17	0.044							0.011	0.238			0.011	0.044	0.370	0.050		
18									0.138				0.431	0.293	0.039	0.006	
19				0.011					0.133				0.359	0.072	0.006	0.028	
20				0.011					0.044				0.122	0.011		0.221	
21				0.193					0.006				0.039	0.006		0.293	
22				0.370					0.006				0.006		0.006	0.116	
23				0.133												0.193	
24				0.099												0.111	
25				0.171												0.017	
26			0.017	0.011												0.017	
27			0.006														
28			0.116														
29			0.254														
30			0.519														
31			0.072														
32			0.011														
33			0.006														
GD	0.6127	0.6184	0.6502	0.7727	0.4779	0.3664	0.7050	0.7225	0.8188	0.6297	0.6779	0.3092	0.6708	0.7389	0.5845	0.8055	0.6498
PIC	0.5492	0.5389	0.5989	0.7362	0.3904	0.3395	0.6426	0.6910	0.7901	0.5582	0.6143	0.2873	0.6076	0.6918	0.5398	0.7729	0.5775
PM	0.3907	0.3851	0.3534	0.2316	0.5247	0.6356	0.2989	0.2815	0.1857	0.3738	0.3258	0.6925	0.3329	0.2652	0.4188	0.1990	0.3537
PD	0.6093	0.6149	0.6466	0.7684	0.4753	0.3644	0.7011	0.7185	0.8143	0.6262	0.6742	0.3075	0.6671	0.7348	0.5812	0.8010	0.6463

Here: GD — gene diversity; PIC — polymorphism information content; PM — probability matching; PD — power of discrimination.

**Table:** Y-chromosome haplotype analysis of seventeen Y-STR markers in male subjects of the Rewa population (n=181)

Haplotype code	Count	DYS19	DYS389I	DYS389II	DYS390	DYS391	DYS392	DYS393	DYS385a	DYS385b	DYS438	DYS439	DYS437	DYS448	DYS458	DYS456	DYS635	Y-GATA-H4
haplo-1	1	13	11	26	24	10	11	13	12	18	10	10	14	17	17	15	21	11
haplo-2	1	14	11	26	24	10	11	12	12	18	10	10	14	17	19	15	22	11
haplo-3	1	14	11	26	24	10	11	13	12	17	10	10	14	17	20	15	22	11
haplo-4	1	14	11	27	23	10	11	13	13	16	9	12	16	21	17	16	23	12
haplo-5	1	14	12	28	23	10	14	12	12	19	11	12	15	20	18	16	21	12
haplo-6	1	14	12	30	23	10	11	13	13	16	10	12	14	19	18	17	25	11
haplo-7	1	14	13	29	22	10	11	12	14	16	11	10	14	19	16	15	22	12
haplo-8	2	14	13	29	23	10	10	14	12	18	11	10	17	19	18	15	24	12
haplo-9	1	14	13	29	23	10	11	12	13	16	9	12	14	20	14	16	21	11
haplo-10	1	14	13	29	24	10	11	13	14	15	10	11	14	18	17	14	20	12
haplo-11	1	14	13	30	24	10	11	12	13	15	9	13	14	18	19	13	21	11
haplo-12	1	14	14	29	25	10	11	13	13	16	10	12	14	19	17	14	22	11
haplo-13	1	14	14	30	22	10	10	14	13	17	12	11	16	18	17	15	26	12
haplo-14	1	14	14	30	22	10	10	14	13	18	12	11	16	18	17	15	26	12
haplo-15	1	14	14	30	23	10	11	12	15	19	9	11	14	20	18	15	23	11
haplo-16	2	14	14	30	23	10	11	13	13	16	9	12	15	21	16	15	23	11
haplo-17	3	14	14	30	23	10	11	13	13	16	9	12	16	21	14	15	24	12
haplo-18	1	14	14	30	23	9	16	13	13	17	11	12	14	22	19	15	22	11
haplo-19	1	14	14	31	22	10	11	12	14	16	9	11	14	19	19	16	19	13
haplo-20	1	14	14	32	24	10	11	13	11	14	11	10	14	17	17	14	23	13
haplo-21	1	15	12	28	22	10	11	12	15	16	9	11	14	19	17	15	20	12
haplo-22	1	15	12	28	22	10	11	12	15	16	9	11	14	19	18	16	20	11
haplo-23	1	15	12	28	22	10	11	12	15	16	9	11	14	19	18	16	21	11
haplo-24	1	15	12	28	22	10	11	12	15	16	9	11	14	19	19	15	21	12
haplo-25	1	15	12	28	22	10	11	12	15	16	9	11	14	19	19	16	21	12
haplo-26	1	15	12	28	22	10	11	12	15	18	9	12	14	19	17	16	20	12
haplo-27	1	15	12	28	22	11	11	12	15	16	9	11	14	19	18	16	20	12
haplo-28	1	15	12	28	22	11	11	12	15	16	9	11	14	19	18	16	21	11
haplo-29	1	15	12	28	23	10	11	12	13	17	9	12	15	18	15	13	21	11
haplo-30	1	15	12	29	22	10	11	12	15	16	9	11	14	19	18	15	20	12
haplo-31	1	15	12	29	26	11	11	13	11	15	11	11	14	20	16	15	24	12
haplo-32	1	15	12	30	22	11	11	12	15	19	9	12	14	19	16	18	20	12
haplo-33	1	15	13	28	22	10	11	15	13	19	10	11	14	19	17	16	20	11
haplo-34	1	15	13	28	22	10	12	12	15	17	10	11	14	19	18	15	20	12
haplo-35	1	15	13	28	23	10	12	12	13	15	10	12	15	19	16	13	21	11
haplo-36	1	15	13	28	24	10	10	13	12	19	11	12	16	20	17	16	24	13
haplo-37	1	15	13	28	24	11	13	13	12	14	12	12	15	18	17	15	24	13
haplo-38	1	15	13	28	25	10	13	14	15	19	10	12	14	18	16	16	21	11
haplo-39	1	15	13	29	20	10	11	12	16	20	8	11	14	19	17	15	20	10
haplo-40	1	15	13	29	21	11	11	12	15	17	9	11	14	19	18	15	20	11
haplo-41	1	15	13	29	22	10	11	12	14	17	9	11	15	19	16	16	20	12
haplo-42	1	15	13	29	22	10	11	12	15	17	9	11	14	19	19	15	21	12
haplo-43	1	15	13	29	22	10	11	12	15	17	9	12	14	19	17	15	20	12
haplo-44	1	15	13	29	22	10	11	12	15	17	9	12	14	19	18	15	20	12
haplo-45	1	15	13	29	22	10	11	12	15	18	9	11	14	19	17	15	19	12
haplo-46	1	15	13	29	22	10	11	12	15	18	9	11	14	19	17	15	20	12
haplo-47	1	15	13	29	22	10	11	12	15	18	9	11	14	19	17	16	20	11
haplo-48	1	15	13	29	22	10	11	12	16	18	9	11	14	19	17	15	19	12
haplo-49	1	15	13	29	22	10	11	13	15	17	9	11	14	18	16	15	21	11
haplo-50	1	15	13	29	22	10	11	13	15	18	9	11	14	18	18	15	21	11
haplo-51	1	15	13	29	22	10	11	13	15	18	9	11	14	19	17	15	19	11
haplo-52	1	15	13	29	22	11	11	12	11	15	10	12	15	18	18	16	21	11
haplo-53	2	15	13	29	22	11	11	12	15	15	10	11	14	19	18	15	24	11
haplo-54	1	15	13	29	24	10	11	12	13	17	9	12	15	19	18	13	20	11
haplo-55	1	15	13	29	24	11	13	14	15	20	10	11	14	18	17	15	21	11
haplo-56	1	15	13	29	25	10	13	12	15	16	11	13	14	18	16	17	20	13
haplo-57	1	15	13	29	25	10	13	12	15	16	14	13	14	18	16	17	20	11
haplo-58	1	15	13	29	25	10	13	13	15	19	10	13	14	17	17	16	21	10
haplo-59	1	15	13	29	25	10	13	14	15	19	10	11	14	18	17	15	21	10
haplo-60	1	15	13	29	25	10	13	14	15	19	10	11	14	18	17	16	18	11
haplo-61	1	15	13	29	25	10	13	14	15	20	10	12	14	18	17	15	22	10
haplo-62	1	15	13	29	25	10	13	14	16	20	10	11	14	18	17	15	21	11
haplo-63	1	15	13	29	25	11	11	15	11	14	11	12	14	19	17	15	22	12
haplo-64	1	15	13	29	25	11	13	13	15	19	10	12	14	19	18	16	21	11
haplo-65	1	15	13	29	25	11	13	14	15	20	10	12	14	18	17	15	20	11
haplo-66	1	15	13	29	25	11	13	14	15	20	10	12	14	18	17	15	21	11
haplo-67	1	15	13	29	25	11	13	15	15	19	10	12	14	18	16	15	21	11
haplo-68	1	15	13	29	25	11	15	14	15	19	10	12	14	18	18	13	21	10
haplo-69	1	15	13	29	25	9	13	13	15	19	10	12	14	18	16	15	20	11
haplo-70	1	15	13	29	26	11	13	14	14	20	10	12	14	18	17	15	21	11
haplo-71	1	15	13	30	21	10	12	12	16	18	9	11	14	19	16	19	20	12
haplo-72	1	15	13	30	22	10	11	12	15	17	9	10	14	19	17	15	20	12
haplo-73	1	15	13	30	22	10	11	12	15	17	9	11	14	19	17	15	19	12
haplo-74	2	15	13	30	22	10	11	12	15	17	9	11	14	19	17	15	20	12
haplo-75	1	15	13	30	22	10	11	12	15	17	9	11	14	19	17	15	21	13
haplo-76	1	15	13	30	22	10	11	13	15	16	9	11	14	18	17	15	21	11
haplo-77	1	15	13	30	22	10	11	13	15	17	9	11	14	18	17	15	20	11
haplo-78	5	15	13	30	22	10	11	13	15	17	9	11	14	18	17	15	21	11
haplo-79	1	15	13	30	22	10	11	13	15	18	9	11	14	18	17	15	21	11
haplo-80	1	15	13	30	22	15	12	12	17	19	11	11	15	19	17	22	20	12
haplo-81	1	15	13	30	23	10	11	12	16	16	10	11	15	19	18	16	20	11
haplo-82	1	15	13	30	23	10	11	12	16	16	9	11	14	19	18	16	20	11
haplo-83	1	15	13	30	25	10	11	13	11	14	11	10	14	20	16	15	24	13
haplo-84	1	15	13	31	19	10	11	13	12	16	10	12	15	18	15	15	22	11
haplo-85	1	15	13	31	19	11	11	13	12	17	10	12	15	17	15	16	22	11
haplo-86	1	15	13	31	22	10	11	14	14	17	10	11	14	18	18	14	21	13
haplo-87	1	15	14	30	20	10	11	13	15	18	9	12	14	18	19	17	20	12
haplo-88	1	15	14	30	21	10	11	12	15	16	10	12	14	18	18	15	21	13
haplo-89	2	15	14	30	21	10	11	15	15	16	10	12	14	18	18	15	21	13
haplo-90	1	15	14	30	21	11	11	13	15	16	10	13	14	18	18	15	24	12
haplo-91	1	15	14	30	22	10	10	12	15	19	9	12	14	19	16	18	20	12
haplo-92	1	15	14	30	22	10	11	12	15	17	9	11	14	19	21	16	20	12
haplo-93	2	15	14	30	22	10	11	12	15	19	9	12	14	19	16	18	20	12
haplo-94	1	15	14	30	22	10	11	12	16	19	9	12	14	19	16	18	20	12
haplo-95	1	15	14	30	22	11	11	14	10	15	11	12	14	20	17	15	22	11
haplo-96	1	15	14	30	23	10	11	12	14	16	10	12	12	19	17	18	22	11
haplo-97	1	15	14	30	24	10	13	11	16	20	10	12	14	18	17	15	21	11
haplo-98	1	15	14	30	24	11	13	14	15	19	10	13	14	17	16	16	21	11
haplo-99	1	15	14	30	25	10	13	14	14	19	10	12	14	18	16	16	21	10
haplo-100	1	15	14	30	25	10	13	14	15	19	10	12	14	18	17	16	20	11
haplo-101	1	15	14	30	25	11	11	14	17	18	10	13	14	18	17	17	22	11
haplo-102	1	15	14	30	25	11	13	13	15	21	10	12	14	18	17	17	21	11
haplo-103	1	15	14	30	25	11	13	14	15	22	10	12	14	18	17	14	21	11
haplo-104	1	15	14	31	21	10	11	14	12	18	10	10	15	19	15	15	21	11
haplo-105	1	15	14	31	23	11	10	12	14	19	11	10	16	19	17	15	26	12
haplo-106	1	15	14	31	24	11	13	14	16	19	10	13	14	18	16	15	22	11
haplo-107	1	15	14	31	25	11	11	13	11	14	11	10	14	20	16	15	23	12
haplo-108	1	15	14	31	25	11	11	13	11	14	11	10	14	20	18	16	23	12
haplo-109	1	15	14	32	25	10	11	13	11	13	12	10	14	20	17	15	23	13
haplo-110	1	15	14	33	24	9	11	12	11	14	11	10	14	20	16	15	23	12
haplo-111	1	16	12	28	23	10	11	13	16	16	10	12	15	20	17	16	24	11
haplo-112	1	16	12	28	23	10	11	13	16	17	10	13	15	20	17	16	24	11
haplo-113	1	16	12	28	23	9	11	12	14	18	10	12	14	19	17	14	21	12
haplo-114	1	16	12	29	23	11	11	12	14	18	10	11	14	19	17	15	21	12
haplo-115	1	16	12	30	21	11	11	14	15	17	10	11	14	18	18	15	23	13
haplo-116	1	16	12	30	22	11	11	13	15	17	10	11	16	21	18	16	22	12
haplo-117	1	16	13	29	21	10	11	13	15	15	10	12	14	18	17	15	21	12
haplo-118	1	16	13	29	22	10	11	12	13	17	10	11	14	18	19	14	21	11
haplo-119	1	16	13	29	22	10	11	13	13	17	10	11	14	18	18	14	21	11
haplo-120	1	16	13	29	25	10	11	13	12	15	11	10	14	20	16	15	23	12
haplo-121	1	16	13	30	22	10	11	12	15	17	9	11	14	19	17	15	20	13
haplo-122	1	16	13	30	22	10	11	13	13	17	10	11	14	18	18	15	22	11
haplo-123	1	16	13	30	22	10	11	13	13	17	10	11	14	18	18	15	23	11
haplo-124	1	16	13	30	22	10	11	13	13	17	10	11	14	18	18	15	24	11
haplo-125	1	16	13	30	22	10	11	13	13	17	10	11	15	18	18	15	23	11
haplo-126	1	16	13	30	22	10	11	13	14	17	10	11	14	18	19	15	23	11
haplo-127	1	16	13	30	22	10	11	14	13	17	10	11	14	18	18	15	23	11
haplo-128	1	16	13	30	22	10	11	14	13	17	10	11	14	18	18	15	24	11
haplo-129	1	16	13	30	24	10	11	14	11	14	11	11	14	20	19	15	21	12
haplo-130	1	16	13	30	25	10	11	13	11	14	11	11	14	20	16	17	24	12
haplo-131	1	16	13	30	25	11	11	13	11	14	11	10	14	20	15	16	23	12
haplo-132	1	16	13	31	25	11	11	13	11	14	11	10	14	20	16	16	23	13
haplo-133	1	16	14	28	21	11	11	15	16	16	10	12	14	18	18	15	22	13
haplo-134	1	16	14	28	21	11	11	15	16	18	10	12	14	18	16	15	22	13
haplo-135	1	16	14	30	21	10	11	12	15	16	9	11	14	20	16	16	21	11
haplo-136	1	16	14	30	21	10	11	14	14	16	10	12	14	18	17	15	23	12
haplo-137	2	16	14	30	21	10	11	14	15	16	10	12	14	18	18	15	23	13
haplo-138	1	16	14	30	21	11	11	14	11	15	12	12	14	20	16	15	22	11
haplo-139	1	16	14	30	21	11	11	14	14	16	10	13	14	18	19	15	21	12
haplo-140	2	16	14	30	21	11	11	14	15	16	10	12	14	18	17	15	23	13
haplo-141	2	16	14	30	21	11	11	14	15	16	10	12	14	18	18	15	23	12
haplo-142	1	16	14	30	21	11	11	14	15	16	10	12	14	18	18	15	23	13
haplo-143	1	16	14	30	21	11	11	14	15	16	10	12	14	18	18	15	24	11
haplo-144	1	16	14	30	21	11	11	14	15	16	10	13	14	18	18	15	22	11
haplo-145	1	16	14	30	21	11	11	14	15	17	10	12	14	18	18	15	23	13
haplo-146	2	16	14	30	21	11	11	14	15	17	10	12	14	18	18	15	25	13
haplo-147	1	16	14	30	21	11	11	14	16	18	10	12	14	18	17	15	23	12
haplo-148	1	16	14	30	21	11	11	15	14	16	10	13	14	18	17	15	21	13
haplo-149	1	16	14	30	21	11	11	15	15	16	10	13	14	18	18	15	23	13
haplo-150	1	16	14	30	21	11	11	15	16	16	10	13	14	18	18	15	23	12
haplo-151	1	16	14	30	21	11	11	15	16	16	10	13	14	18	18	15	23	13
haplo-152	1	16	14	30	22	10	11	12	14	15	10	10	14	19	18	16	24	10
haplo-153	1	16	14	30	22	10	11	12	15	18	9	11	14	19	16	17	20	12
haplo-154	1	16	14	30	22	10	11	12	15	18	9	11	14	19	16	18	20	11
haplo-155	1	16	14	30	25	9	11	13	11	14	11	10	14	19	17	16	23	11
haplo-156	1	16	14	31	25	11	11	13	13	16	9	11	14	19	16	15	23	12
haplo-157	1	16	15	31	21	11	11	14	15	16	10	14	14	18	18	15	24	11
haplo-158	1	17	12	30	24	10	10	13	13	14	9	12	14	20	19	14	22	11
haplo-159	1	17	13	30	21	10	11	14	15	18	10	12	15	19	20	16	23	11
haplo-160	1	17	13	30	21	10	11	14	16	17	10	12	15	19	17	16	22	11
haplo-161	1	17	13	30	23	11	11	13	12	18	10	11	15	19	15	17	21	12
haplo-162	1	17	13	31	24	11	11	13	11	14	11	10	14	20	14	15	23	12
haplo-163	1	17	14	30	22	10	11	12	14	15	10	14	14	19	17	16	21	12
haplo-164	1	17	14	30	22	10	11	12	14	16	10	13	14	19	16	16	20	12
haplo-165	1	17	14	30	24	11	13	14	13	19	10	13	14	17	17	16	23	12
Total number of haplotypes = 181																		
Number of obtained haplotypes = 165																		
Number of unique haplotypes = 153																		
Haplotype diversity = 0.9985																		
Discriminating capacity = 0.927																		
